# Up and down the spectrum: upconversion nanocrystal and semiconductor material fused into a single nanocomposite

**DOI:** 10.1038/s41377-022-00875-9

**Published:** 2022-06-14

**Authors:** Hans H. Gorris, Zdeněk Farka

**Affiliations:** Department of Biochemistry, Masaryk University, 625 00 Brno, Czech Republic

**Keywords:** Nanoparticles, Fluorescence spectroscopy

## Abstract

A nanocomposite consisting of a cubic EuSe semiconductor material grown on a hexagonal upconversion nanoparticle has overcome the crystal lattice mismatch that typically prevents the epitaxial growth of such heterogeneous nanocrystals. Eu^3+^ at the interface layer shows its characteristic red emission band both under UV excitation light due to energy transfer from the semiconductor and under NIR excitation light due to energy transfer after photon-upconversion. Data storage and security applications are suggested for this new nanocomposite.

## Abstract (web summary)

A colorful approach to tackle the lattice mismatch in a nanocrystal

## Graphical abstract

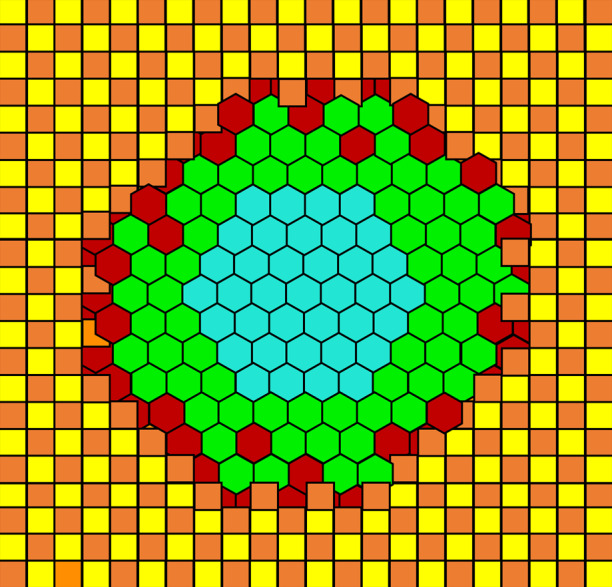

The conversion of invisible ultraviolet (UV) light or near-infrared (IR) radiation into visible light is a challenging task that can be accomplished by exploiting the rich ladder-like energy states of lanthanide ions. Due to similar ionic radii, lanthanides can be replaced in a crystalline host matrix by one another, added in different combinations and in different concentrations without strongly affecting the crystal structure. It is thus in principle possible to assemble the full spectral range of different lanthanide ions into a single homogeneous nanocrystal. Furthermore, numerous energy transfer processes occur concurrently among different lanthanide ions in the host crystal, which lays the foundation for energy transfer upconversion (ETU). One of the best-known examples is the absorption of two or more photons of 980-nm NIR light by Yb^3+^ with subsequent energy transfer steps to Tm^3+^, which leads to the characteristic emission of blue (and 800-nm NIR) light. Such upconversion nanomaterials enable a wide range of nanophotonic applications in biomedical diagnosis, immunoassays, imaging, temperature sensing, green energy conversion, data storage, and anti-counterfeiting^1^.

Some energy transfer processes in the host matrix such as cross relaxation, however, are destructive and must be eliminated. Thus, over the last 15 years many core-shell architectures have been designed to confine different lanthanide ions in different compartments of the nanoparticles to avoid destructive interference or to enhance specific optical features^2^. The epitaxial growth of a shell can be repeated several times to obtain multiple layers of different lanthanide compositions. Although each layer has a distinct lanthanide composition, they all share the same crystalline matrix. Alternatively, similar ionic radii of lanthanide ions and Ca^2+^ facilitate a heteroepitaxial growth of a CaF_2_ shell on NaYF_4_ nanoparticles—or vice versa^3^. In this case, both the core and the shell typically have the same cubic (*α*) crystal phase to keep the lattice mismatch minimal. This is, however, not the best combination as the hexagonal structure is a much more efficient upconversion material than the cubic structure^4^. It should also be noted that CaF_2_ layers were mainly developed as (optically) passive shells to avoid surface quenching effects or to improve the biocompatibility rather than being directly involved in energy transfer processes.

Sun and Bednarkiewicz^5^ now made a significant step forward by growing the cubic semiconductor material EuSe on a hexagonal core/shell nanocrystal (NaLnF_4_, Fig. 1a). They achieved this by modifying the interface in an ingenious way: In a first step, some of the surface lanthanide ions (Ln^3+^) of the NaLnF_4_ nanocrystal were replaced by Eu^3+^, which - as a trivalent lanthanide ion - does not interfere with the crystal structure of the host matrix. In a subsequent reduction step, oleyl amine was used to partially reduce Eu^3+^ to Eu^2+^, which constituted an optimal interface to overcome the lattice mismatch and enabled the epitaxial growth of a cubic Eu(II)Se shell on the hexagonal NaLnF_4_ nanocrystal.

**Fig. 1:**
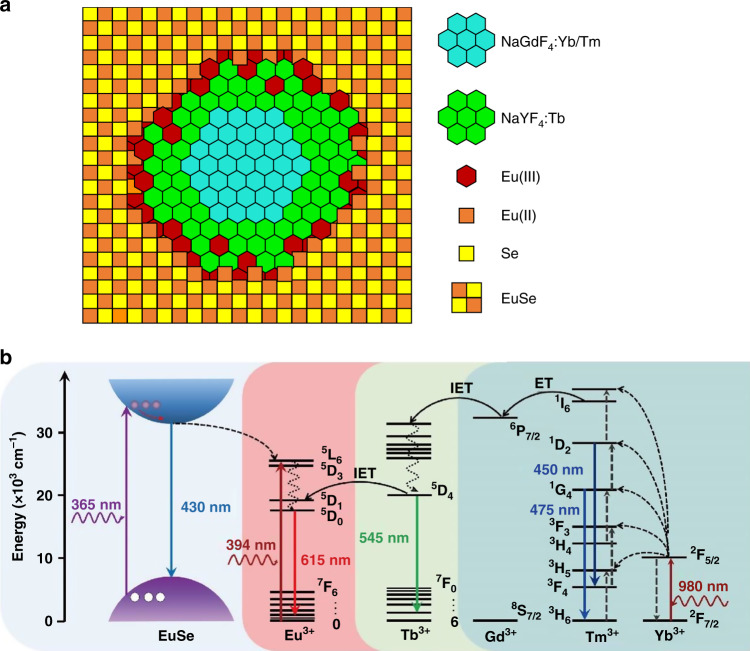
Upconversion nanocrystal and semiconductor material fused into a single nanocomposite. **a** Epitaxial growth of the cubic semiconductor EuSe on hexagonal NaYF_4_ is facilitated by replacing some surface lanthanide ions in the NaYF_4_ host matrix by Eu^3+^, which is then reduced to Eu^2+^. **b** Energy transfer steps in the nanocomposite under UV (365 and 394 nm) excitation, which yields Stokes luminescence, and under NIR (980 nm) excitation, which yields anti-Stokes luminescence

In this novel nanocomposite design, Eu^3+^ takes center stage because the population of high energy states in Eu^3+^ and the concomitant emission of red light (615 nm) can be fed from three light sources (Fig. 1b): First, Eu^3+^ absorbs UV light (394 nm) directly. Second, the semiconductor EuSe absorbs UV light of shorter wavelength (365 nm) and passes this energy on to Eu^3+^. Third, after the upconversion process in the core particle (NaGdF_4_:Yb/Tm), excitation energy is transferred from Tm^3+^ over Gd^3+^ to Tb^3+^ in the NaYF_4_:Tb shell and finally to Eu^3+^ at the interface layer. As these energy transfer steps additionally yield lanthanide-specific emission lines, the multicolor emission of Tm^3+^ (blue), Tb^3+^ (green) and Eu^3+^ (red) appears as white light.

This dual-mode luminescent nanocomposite provides a promising blueprint for the design of a wide range of heterostructures with tailor-made optical properties. In addition to information storage and anticounterfeiting, these nanocomposites may be particularly useful for multiplexed applications in diagnostics and imaging^6^. Further applications will strongly benefit from improving the quantum yields both in the upconversion mode (<1%) and in the down-shifting mode (5%). If the quantum yields are improved, these heterostructures may even become valuable for light harvesting applications^7^.
